# Research on the site selection of emergency medical facilities from the perspective of country parks

**DOI:** 10.1038/s41598-023-47610-x

**Published:** 2023-11-24

**Authors:** Zheng Wu, Shuai Li, Xiangxu Li, Jiefang Tang, Jiangtao Jiu, Pengfei Wang

**Affiliations:** 1 College of Landscape Architecture and Art, Henan Agricultural University, Zhengzhou, 450002 China; 2 College of Architecture and Urban Planning, Tongji University, Shanghai, 200092 China

**Keywords:** Natural hazards, Public health

## Abstract

Post-epidemic era, human society entered the stage of epidemic prevention and control normalized, emergency medical facilities are an important means of epidemic prevention and control, attention-needed can provide construction sites for emergency medical facilities. Disaster prevention and green space can provide construction sites for emergency medical facilities. In comparison, it was found that the site selection conditions of country parks and emergency medical facilities were more compatible. Based on the requirement of the latter's location, using the Delphi method and analytic hierarchy process, through to the country park type, effective safety area, space fragmentation, distance away from the water, in the wind, away from the city center distance, hydrogeology, eight factors such as traffic is quantitative, construct the evaluation system of emergency medical facility location. Taking Nanjing as an example, the Nanjing city region within the scope of the 43 country parks comprehensive comparison. Results show that green hill forest park, the highest scores in addition to the traffic time factor, the remaining seven factor score the highest, the most balanced distribution, security, scalability, rehabilitation, convenience, pollution prevention, and evacuation. It can be preferred as the site selection and construction target of emergency medical facilities. Besides, the Youzishan Forest Park and Dongkeng Country Park comprehensive scores and each factor score is higher, can be used as emergency preplan alternative goals. The score results indicate that the evaluation method for severity exhibits higher levels of differentiation, significant validity, and highly consistent assignment of impact factor weights. In view of the different regional land, impact factor weights assignment can be adjusted adjust measures to local conditions, to effectively make use of the existing conditions, avoid adverse factors.

## Introduction

In the post-epidemic era, COVID-19 will coexist with human society for a long time, with sporadic and seasonal outbreaks. Human society has entered a normalized stage of epidemic prevention and control. Since the outbreak of COVID-19 in Wuhan, China in 2020, the pandemic has spread to more than 200 countries and regions around the world^1^, and is defined by the World Health Organization (WHO) as a serious global public health emergency^2^. The number of confirmed cases is increasing exponentially, posing a huge threat and challenge to the global public health and safety system^3, 4^. Inadequate supply of medical facilities, shortage of medical resources, and difficulty in receiving medical treatment in multiple places. The key to responding to large-scale infectious disease outbreaks is to improve admission and cure rates^5^. When existing treatment hospitals are unable to meet the treatment needs, building emergency medical facilities can play a crucial role in responding to the pandemic. Therefore, the location selection of emergency medical facilities is an important research issue in formulating emergency management plans.

In 2003, the SARS epidemic broke out in Beijing, China. In order to control the further development of the SARS epidemic, the Beijing government decided to build a temporary field hospital in *Xiaotangshan* to fight against the SARS epidemic^6^. After the outbreak of COVID-19, the Chinese government quickly built *Leishenshan* Hospital and *Huoshenshan* Hospital in Wuhan to treat confirmed cases to control the further spread of the virus^7^. As the epidemic spreads, other countries such as Russia, South Korea, Italy, Israel and the United States have also begun building emergency medical facilities to deal with the new coronavirus epidemic^8–11^. Building emergency medical facilities has proven to be very useful in cities' fight against the pandemic, aiding in the treatment and recovery of infected people, significantly reducing pandemic-related deaths and curbing the spread of the virus^12^.

As one of the important components of urban public space, urban green space plays a positive role in improving urban adaptation to climate change and responding to the challenges brought by COVID-19^13^. Urban green spaces (UGS) can also provide the public with opportunities for human interaction, exercise, stress relief, and contact with nature, resulting in comprehensive economic, ecological and social benefits^14^. After the outbreak of the pandemic, many scholars studied the relationship between urban green space or urban green space and the COVID-19 epidemic. For example, Jordi Honey Rosés and others suggested that in the COVID-19 pandemic, the existing types and functions of green space should be re-examined^15^. Yang et al. emphasized the positive impact of UGS on public health and found that people living in environments with more green vegetation have less decline in physical activity levels compared to those living in environments with less green vegetation^16^. and green spaces have inhibitory effects on the spread of SARS-CoV-2 virus^17^. In addition, a study by Yao et al. found that during the COVID-19, the use of park green space was positively correlated with mental health^18^. Maury Mora et al. conducted a survey and confirmed that the interaction between indoor plants cannot replace different outdoor green experiences when people face stress situations^19^. Therefore, we believe that emergency medical facilities should be built in urban green space or green public space, and good landscape and plant configuration can help patients recover faster and relieve the psychological pressure of medical staff^20^. When a pandemic occurs, people tend to choose to stay at home rather than go outdoors, which also leads to low utilization of urban green space during the pandemic period^21^. From the perspective of urban land use, constructing emergency medical facilities in urban suburban parks can help alleviate the land shortage faced during urban expansion.

In summary, the selection of emergency medical facilities should take into account both the general requirements of comprehensive hospitals and their unique characteristics. Therefore, this study will compare various types of park green spaces in the city based on the requirements and standards of emergency medical facility construction, and find that the site selection conditions for country parks and emergency medical facilities are highly compatible. Especially during the pandemic, the COVID-19 outbreak was serious in some major cities, and the green space system construction in these cities was relatively complete, especially the number of country parks was large. Therefore, this article takes Nanjing suburban parks as an example and uses AHP and Delphi methods to construct a site selection evaluation system. It quantifies and compares the factors that affect the site selection of emergency medical facilities, and selects suitable suburban parks for constructing emergency medical facilities to provide implementable emergency management measures to respond to the arrival of the pandemic.

## Research objects and methods

### Overview of the research area

Following the end of the first round of COVID-19 in Wuhan, China, Beijing, Guangdong, Jiangsu and other places have witnessed the spread of the epidemic caused by imported cases from abroad, which spilled over to many surrounding areas. In July 2021, a local outbreak caused by an imported case of flight CA910 from Russia broke out in Nanjing and quickly spread to more than ten provinces and municipalities directly under the central government, including Hunan, Anhui, Guangdong, Sichuan, Chongqing, and Hubei. It was the most widespread local outbreak since the outbreak in Wuhan.

**Figure 1:**
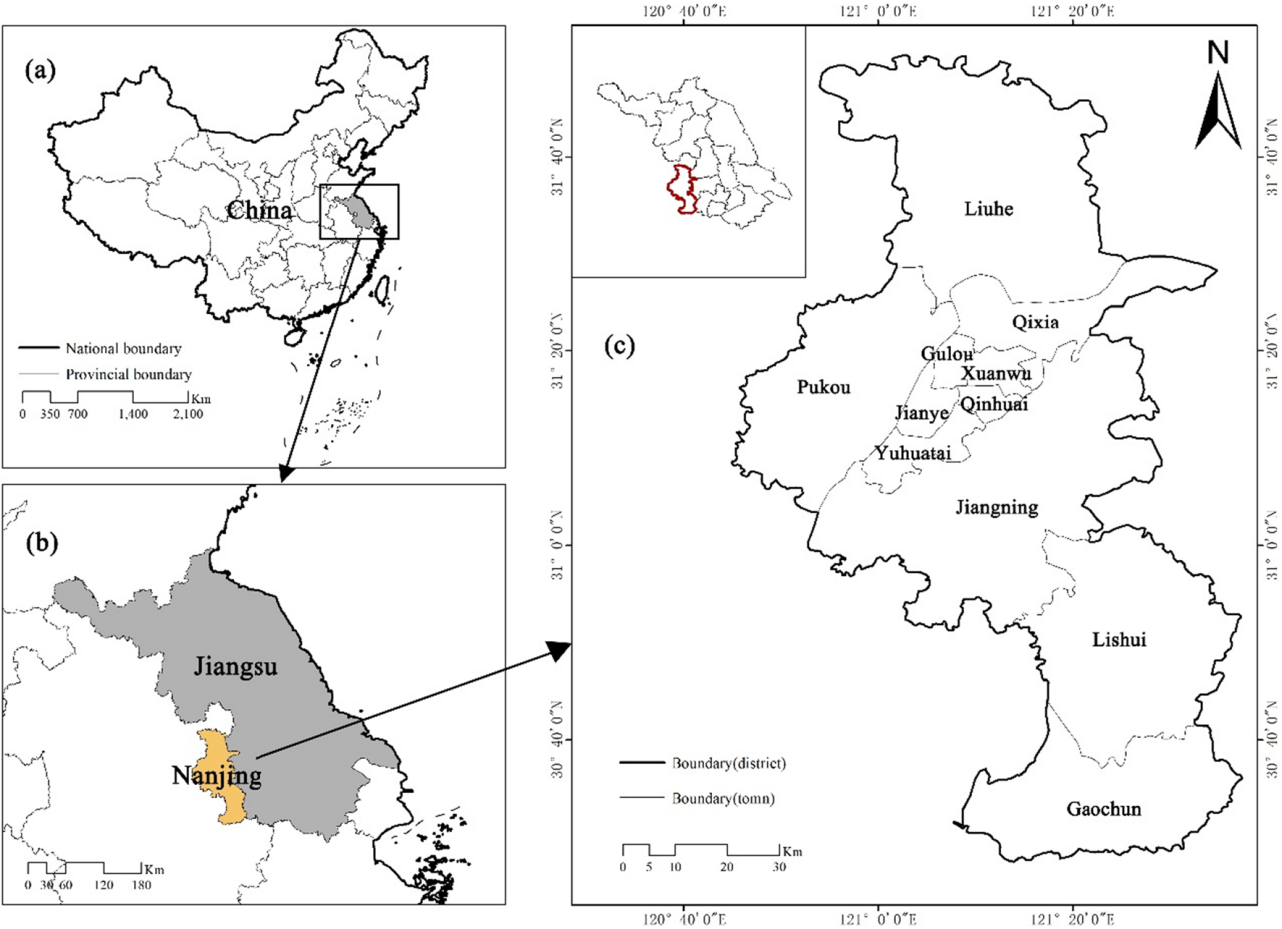
Location of Nanjing City (The map was generated using ArcGIS 10.8 (https://www.esri.com/en-us/home) based on China Standard Map (http://bzdt.ch.mnr.gov.cn/)).

Nanjing abbreviated as "Ning" and formerly known as Jinling and Jiankang, is the capital of Jiangsu Province, a sub-provincial city, a mega city, and a core city in the Nanjing metropolitan area. It has been approved by the State Council as an important central city in eastern China, an important research and education base, and a comprehensive transportation hub in China. The urban area of Nanjing is 6587.02 km^2^, with a permanent population of 9.4911 million and an urban population of 8.258 million. The urbanization rate is 87.01%. Nanjing is an important city in eastern China and an important comprehensive transportation hub in China. The Nanjing railway hub has built a "meter" shaped high-speed railway network directly connecting China, forming a one-hour high-speed rail traffic circle with Shanghai, Hangzhou and Hefei, and a 3–5 h high-speed rail traffic circle with Beijing, Tianjin, Jinan, Zhengzhou, Wuhan, Nanchang, Mount Huangshan, Ningbo, Qingdao, Fuzhou, etc. The radiation center function of Nanjing's railway hub has been further strengthened. Improper epidemic prevention and control in Nanjing can easily lead to the outbreak and spillover of imported epidemics. Therefore, it is imperative to develop corresponding contingency plans in Nanjing (Fig. 1).

## Research objects and data sources

**Figure 2:**
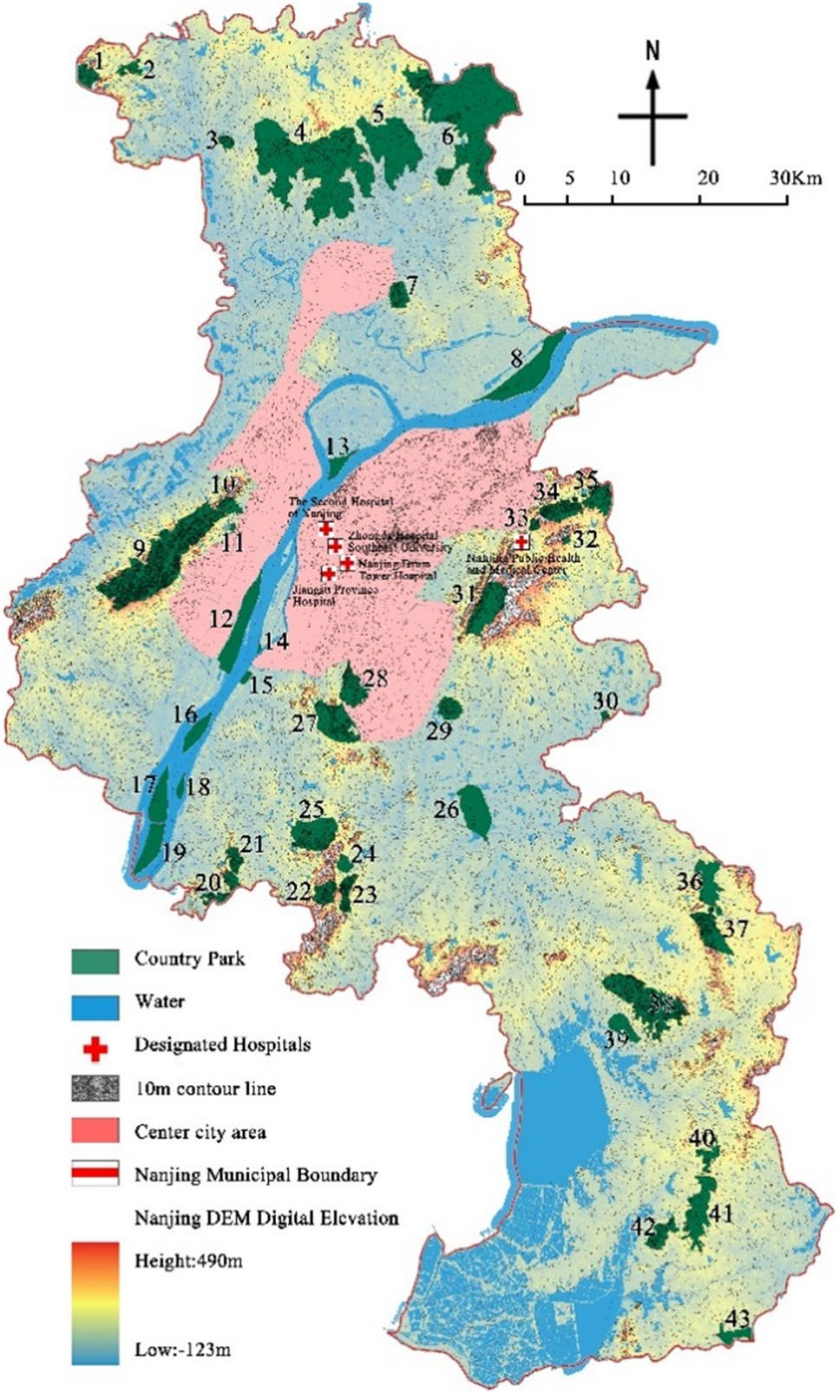
Distribution map of country parks in Nanjing (The map was generated using ArcGIS 10.8 (https://www.esri.com/en-us/home) based on China Standard Map (http://bzdt.ch.mnr.gov.cn/)).

District in Nanjing city as the research scope, and combined with the implementation opinion on strengthening the construction of country park, the city of Nanjing accelerate the construction of ecological civilization action plan (2015–2017) for three years, the Nanjing city green space system planning (2013–2020) and the country park, Nanjing city under the background of ecological civilization construction thought in the designated 43 country parks as the research object of the base(Table 1). Meanwhile, by referring to the Omap interactive map system, Google Map satellite image, and Baidu map system, the location, area, and other information of 43 country parks were corrected by using the digitalization function of the registration domain of ArcGIS10.5 (Fig. 2).

**Table 1:** Statistics of country parks of Nanjing.

Park number	Park name	Construction scale (hm^2^)	Park type
1	Zhimaling Forest Park	495.99	Mountain type
2	Bamboo Town Forest Park	293.34	Forest type
3	Panshan Country Park	210.71	Mountain type
4	Pingshan Forest Park	8783.51	Mountain & Forest type
5	Guizi Mountain Stone Column Forest Scenic Spot	3173.91	Humanistic type
6	Jinniu Lake Scenic Spot	8437.00	Wetland type
7	Lingyan Mountain Country Park	544.10	Mountain & Forest type
8	Longpao Wetland Park	2162.12	Wetland type
9	Laoshan Forest Park	5659.55	Mountain & Forest type
10	Pearl Spring Scenic Spot	370.74	Wetland type
11	Fohand Lake Country Park	49.98	Wetland type
12	Lvshuiwan Ecological Wetland Park	1633.72	Wetland type
13	Baguazhou Wetland Park	382.79	Wetland type
14	Jiangxinzhou Yuantoushi Wetland Park	37.09	Wetland type
15	Sanqiao Binjiang Country Park	116.03	Wetland type
16	Zimuzhou Wetland Park	549.12	Wetland type
17	Xinjizhou Wetland Park	901.58	Wetland type
18	Zihuizhou Wetland Park	158.33	Wetland type
19	Xinshengzhou Wetland Park	933.24	Wetland type
20	Nanshan Forest Park	605.89	Mountain type
21	Baitou Mountain Forest Park	373.09	Mountain type
22	Dongkeng Country Park	542.82	Mountain type
23	Yuntai Mountain Country Park	505.75	Mountain type
24	Ganquan Lake Country Park	265.18	Wetland type
25	Ginkgo Lake Country Park	1562.16	Wetland type
26	Qinhuai River Wetland Park	1443.89	Wetland type
27	Niushou Zutangshan Country Park	1551.86	Mountain type & Humanities type
28	Jiangjunshan Park	933.50	Mountain type
29	Fangshan Country Park	561.32	Mountain type & Humanities type
30	Chishan Country Park	95.98	Mountain type
31	Qinglongshan Country Park	1476.57	Mountain type
32	Tangshan Mine Country Park	66.18	Mountain type
33	Yangshan Beicai Country Park	131.53	Humanistic type
34	Tangshan Xianrenqiao Country Park	846.61	Mountain type
35	Anji Shanshui Reservoir Country Park	847.94	Mountain type
36	Dongping Lake Wetland Park	1121.52	Wetland type
37	Donglu Mountain Scenic Area	1114.50	Mountain type & Humanities type
38	Wuxiang Mountain Forest Park	3082.32	Mountain type
39	Fujiabian Nonjiale Country Park	619.40	Pastoral type
40	Dajingshan Forest Park	430.00	Mountain type
41	Qingshan Forest Park	1602.98	Forest type
42	Youzishan Forest Park	661.23	Mountain type
43	Fujiatan Forest Park	503.42	Forest type

## Research methods

Firstly, by reviewing the site selection requirements and construction standards for infectious disease hospitals and emergency medical facilities, we found that since water bodies are important vectors for the transmission of pathogenic microorganisms and pose a risk of spreading infectious diseases, we should choose places that are far from the water source and have relatively flat terrain^22^. The lower limit of emergency medical facility capacity should be considered to ensure sufficient emergency area^23^. Emergency medical facilities require a continuous and single site, so it is necessary to choose a location with low fragmentation. The wind direction of a city can also affect the site selection, and wind is also one of the vectors of virus transmission. To avoid the spread of the epidemic caused by wind, emergency medical facilities should be built downwind of the city^24, 25^. Infectious disease hospitals or emergency medical facilities should be built in areas far from urban centers and densely populated areas. At the same time, in order to ensure timely transportation of confirmed patients, the transportation time should be as short as possible^26, 27^. In addition, medical waste may carry pathogens. To prevent these harmful substances from seeping into the ground, sites with high terrain, low groundwater level, and good impermeability should be selected^28, 29^. Therefore, we consider park type, effective risk avoidance area, space fragmentation, distance from water source, wind direction, distance from central urban area, hydrogeological profile and traffic duration of the country parks as eight factors that affect the location of emergency medical facilities.

Secondly, through the Analytic Hierarchy Process (AHP) and Delphi method to various influence factors of empowerment^30^: according to the requirement, each influence factor to the list, and pooling expertise through E-mail way, through after several rounds of feedback, will collect the opinions of the experts put forward is concentrated (Table 2).

**Table 2:** Weight table of influence factors.

Influence factor	Park type (*w*_*1*_)	Effective safe haven area(*w*_*2*_)	Space fragmentation(*w*_*3*_)	Distance from water source(*w*_*4*_)	Wind direction(*w*_*5*_)	Distance from the central city area(*w*_*6*_)	Hydrogeological overview(*w*_*7*_)	Traffic duration(*w*_*8*_)	Total
weight	12	8	12	20	8	16	12	12	100

Thirdly, all influencing factors are assigned by five level system, from inferior to superior in turn into I, II, III, IV, V five grades, the corresponding score is 1, 2, 3, 4, 5 points. According to the actual situation of each country park, through natural breakpoint method of each influence factor classification assignment, then it is worth points (*f*_*i*_) and the corresponding weights (*w*_*i*_) Multiply to get the score of the impact factor (*y*_*i*_), the scores of impact factor of each impact factor score and then weighted and calculation, and obtain the comprehensive rating of the country park (*Y*). The formula is as follows:

1 $$\sum\limits_{i = 1}^{8} {w_{i} } = 100$$

2 $$y_{i} = w_{i} f_{i}$$

3 $$Y = \sum\limits_{i = 1}^{8} {w_{i} f_{i} }$$

4 $$Y = 12f_{1} + 8f_{2} + 12f_{3} + 20f_{4} + 8f_{5} + 16f_{6} + 12f_{7} + 12f_{8}$$

Fourth, the 43 country parks were given a comprehensive score and ranked in order. The country parks with high scores were more suitable sites for emergency medical facilities than those with low scores.

## Impact factor value assignment method

### Park type (***f***_***1***_)

Nanjing is located in the western section of the Ningzhen Mountain Range. The Yangtze River runs through the area from southwest to east. The overall terrain and landform gradually transition from the low hills in the southwest and northwest to the Yangtze River valley plain in the middle. It can be divided into four types: mountain, hill, upland and plain^31^.

Taking into account the construction theme, surface form and resource types of country parks, the 43 country parks types are classified, and their types can be divided into five types: wetland country parks, humanistic country parks, mountainous country parks, idyllic country parks and forest country parks. First of all, water is an important medium for spreading diseases after ingestion or contact with pathogenic microorganisms, such as cholera, hepatitis A, typhoid fever, etc. Eight of the 37 statutory infectious diseases in China are all waterborne infectious diseases^22^. The wetland type country parks are close to water sources, and if they are not properly protected, they will be easily affected by them, so they are classified as Class I. The humanistic landscape is a non-renewable resource, and Nanjing enjoys the reputation of "ancient capital of six dynasties" and "capital of ten generations", with nearly 2500 years of city history and about 450 years of capital history and rich cultural and material relics above and below ground. Therefore, the humanistic type park is set as Class II; the mountainous type country park mainly contains uncertainties in terms of traffic and site topography and geological conditions, and is set as Class III; the idyllic type country park has flat topography, convenient transportation, high integration of land parcels and relatively complete infrastructure, and is set as Class IV. Forested country parks are open and have excellent ecological environment. Large areas of dense forest can play a role of isolation and provide a good forest rehabilitation environment for patients^32^, so this type of country park is set as Class V.

### Effective safe haven area(***f***_***2***_)

The siting of emergency medical facilities should take into account the lower limit of effective safe haven area. 2020 With the outbreak of New Crown Pneumonia, the first nationwide major public health emergencies level I response was launched. Referring to the construction and use of *Xiaotangshan*, *Huoshenshan* and *Leishenshan* hospitals, the data of the New Crown Pneumonia epidemic is used as the basis, i.e., when the emergency medical facility is a single-story building, its effective land area should be no less than 4.7 hm^2^^33^. The effective shelter area in the urban disaster prevention and avoidance function green space refers to the actual area that can be used for disaster prevention and avoidance after deducting the area of water, building (structure) and its falling objects and collapse influence (the radius of the influence area is calculated by 50% of the height of the building (structure)), the dense area of trees, the area of slope greater than 15% and the rescue channel, etc. It is also stipulated that the effective shelter area of the country park as a long-term shelter green space is not less than 30 hm^2^. According to the statistics of Nanjing country parks, 43 country parks with effective refuge areas ranging from 17.62 to 8110.85 hm^2^ are in line with the lower limit of land requirement for emergency medical facilities. The country parks with an effective shelter area of 4.7–30 hm^2^ are classified as Class I, between 30 and 150 hm^2^ as Class II, 150–750 hm^2^ as Class III, 750–3750 hm^2^ as Class IV, and > 3750 hm^2^ as Class V.

### Spatial fragmentation(***f***_***3***_)

Spatial patchiness is the most common form of landscape pattern, reflecting the heterogeneity of the landscape; fragmentation characterizes the degree of fragmentation of the park by water systems, roads, structures, etc., reflecting the complexity of the park's spatial structure^34^. Emergency medical facilities require a single, homogeneous and continuous building site within the park, so parks with a low number of spatial patches and low landscape fragmentation are more suitable as target sites. Landscape fragmentation^35^. The ratio of the number of patches to the corresponding area is usually used, i.e. *C*_*i*_ = *N*_*i*_/*A*_*i*_. The larger the fragmentation degree, the more fragmented the internal space of the park is, and the smaller the area of effective risk-avoidance patches is, which is not conducive to the construction of emergency medical facilities and later expansion. The statistical results showed that the spatial fragmentation of 43 country parks ranged from 0.0003 to 0.1703. According to the fragmentation distribution characteristics, the natural interruption point method was used to classify them, so 0.0003–0.0047 was class V, 0.004701–0.0120 was class IV, 0.012001–0.0240 was class III, 0.024001–0.0511 was level II, 0.051101–0.1703 for level I.

### Distance from water source(***f***_***4***_)

Nanjing is a riverside port city straddling both sides of the Yangtze River. The water area of Nanjing accounts for about 11% of the city, and there are three water systems in its territory: the Yangtze River, the Huai River, and Taihu Lake, of which the Yangtze River system is the main water system in Nanjing, which can be subdivided into four sub-water systems, including the Chu River, the Yangtze River along the Nanjing River, the Qinhuai River, and the Suiyang River, involving all districts and counties of Nanjing, with a watershed area of 6287.7 km^2^, accounting for 95.49% of the total land area of Nanjing. The above water systems enter the study area to form various types of surface water sources such as rivers, lakes, wetlands and reservoirs. In the “technical specifications for the delineation of drinking water source protection zones”, different types of water sources such as rivers, lakes, reservoirs and water protection zones are delineated in the land area of protection, of which the scope of protection for large reservoirs is most strictly defined. River-type water sources, primary and secondary land area protection range to the coastal depth and the horizontal distance of the river bank not less than 50 m and 1000 m for the boundary. Lakes, reservoirs type water sources, primary protection including lakes, reservoirs, water intake side of the normal water level above the 200 m land area; secondary protection, and according to the scale of lakes, reservoirs are divided into "not less than 3000 m outside the primary protection zone", "above the normal water level (the first protection zone (outside the primary protection zone), the horizontal distance of 2000 m area" two division methods. Combined with the characteristics of the type of water sources within the city of Nanjing, in accordance with the principle of strict division, the above division boundaries integration, set 200 m within the land area (including water sources) for the I level, 200–500 m for the II level, 500–1000 m for the III level, 1000–2000 m for the IV level, > 2000 m for the V level.

### Wind direction(***f***_***5***_)

In the Guidelines for the Site Selection, Design, Construction and Operation Management of Emergency Infectious Disease Hospitals, it is stated that the site selection of emergency medical facilities should be located in the direction of the prevailing downwind of the urban area all year round. Nanjing belongs to the northern subtropical humid monsoon climate zone, which is influenced by the Eurasian continental air mass in winter with northeasterly winds, and by the Eurasian low pressure area in summer with southeasterly winds. The wind frequency calculation method of 16 rows of longitude points was used to quantitatively evaluate the wind frequencies in each direction in Nanjing, centering on the core area. The meteorological data of Nanjing were counted, and the wind frequencies of its sixteen wind directions are shown in Table 3. The wind frequencies in each direction were mainly distributed below 12%, and with reference to the natural intermittent point method grading, they were divided into five frequency bands of 10.44–11.36%, 9.02–9.27%, 6.02–6.73%, 3.68–4.45%, and 2.99–3.26%, and the parks in the upwind area corresponding to the wind direction were assigned as I, II, III, IV, and V in turn.

**Table 3:** Annual frequency of wind directions in Nanjing (%).

Wind direction	N	NNE	NE	ENE	E	ESE	SE	SSE
Frequency	6.73	6.02	11.36	11.3	10.44	9.02	9.27	6.48

Wind direction	S	SSW	SW	WSW	W	WNW	NW	NNW
Frequency	4.19	2.99	3.68	3.76	3.95	3.1	3.26	4.45

### Distance from the central city area(***f***_***6***_)

The Guidelines for the Selection, Design, Construction and Operation of Emergency Infectious Disease Hospitals and the Architectural Design Guidelines for Hospitals for Patients with Infectious Atypical Pneumonia both state that “avoiding densely populated urban areas” is an important principle for site selection. According to the distribution characteristics of country parks in Nanjing, the boundary of Nanjing central city planning area is used as the starting boundary, and the area is divided into I, II, III, IV and V levels from near to far with 15 km as the first level.

### Hydrogeology(***f***_***7***_)

Medical waste carries a large number of pathogens, heavy metals and organic pollutants, which can produce a variety of harmful leachate after rainwater and biological hydrolysis. In order to avoid the harmful leachate from entering the soil with the rainwater and thus causing pollution to the groundwater, emergency medical facilities are usually built on sites with high terrain and low groundwater level and not easy to infiltrate. The stratigraphy of Nanjing belongs to the lower Yangzi subzone of the Yangzi stratigraphic region, and the deep groundwater in the region can be divided into three major types: loose rock pore water, carbonate karst water and clastic rock fracture water^36^. The hydrogeological profile of the 43 study sites was analyzed, and country parks located in potential inundation areas were classified as Class I. Those located in areas with exposed carbonate fissure water and exposed carbonate fissure water cover with water enrichment > 1000 m^3^/d were classified as Class II. Those located in areas with intrusive magmatic fissure water, magmatic fissure water ejected rock, loose rock pore water, clastic rock Fissure water, carbonate fissure water exposed rock, carbonate fissure water exposed class cover and the degree of water richness in the area of 100–1000 m^3^/d is set as level III, located in the magmatic rock fissure water ejected rock, clastic rock fissure water and the degree of water richness < 100 m^3^/d and loose rock pore water and the degree of water richness in the area of 10-100 m^3^/d is set as level IV, located in loose rock pore water and the degree of water richness in the area of < 10 m^3^/d is set as level III. Water and water-rich degree in the < 10 m^3^/d area is set to level V.

### Traffic duration(***f***_***8***_)

During the outbreak of the epidemic, according to the "five centralized" treatment principle of COVID-19, a large number of critically ill patients need to be transferred from the designated treatment institutions in the urban area to the emergency medical facilities through negative pressure ambulance, so the speed of traffic is very critical. During the COVID-19 outbreak, there were 5 designated COVID-19 treatment hospitals set up in Nanjing (Table 4). In the same period, the traffic time of each country park was measured by the digital map navigation system in driving mode, using the above five designated hospitals as the starting point, and the longest traffic time was evaluated according to the principle of strict evaluation. The longest traffic time in the 43 country parks was about 2.5 h. The traffic duration was divided into five periods: ≥ 105 min, 90–105 min, 75–90 min, 60–75 min, and < 60 min, which were divided into I, II, III, IV, and V levels in order.

**Table 4:** Designated hospitals for COVID-19 treatment in Nanjing.

No	List of hospitals	Treatment level	Address
1	Jiangsu Provincial People's Hospital	provincial level	No.300, Guangzhou Road, Gulou District
2	Zhongda Hospital of Southeast University	provincial level	Gulou District, Dingjiaqiao, No.87
3	Nanjing Gulou Hospital	city level	No.321, Zhongshan Road, Gulou District
4	Nanjing Second Hospital	city level	No.1–1, Zhongfu Road, Gulou District
5	Nanjing Public Health Medical Center	city level	Jiangning District Tangshan Street rehabilitation Road No.1

## Evaluation of impact factors and analysis of results

**Figure 3:**
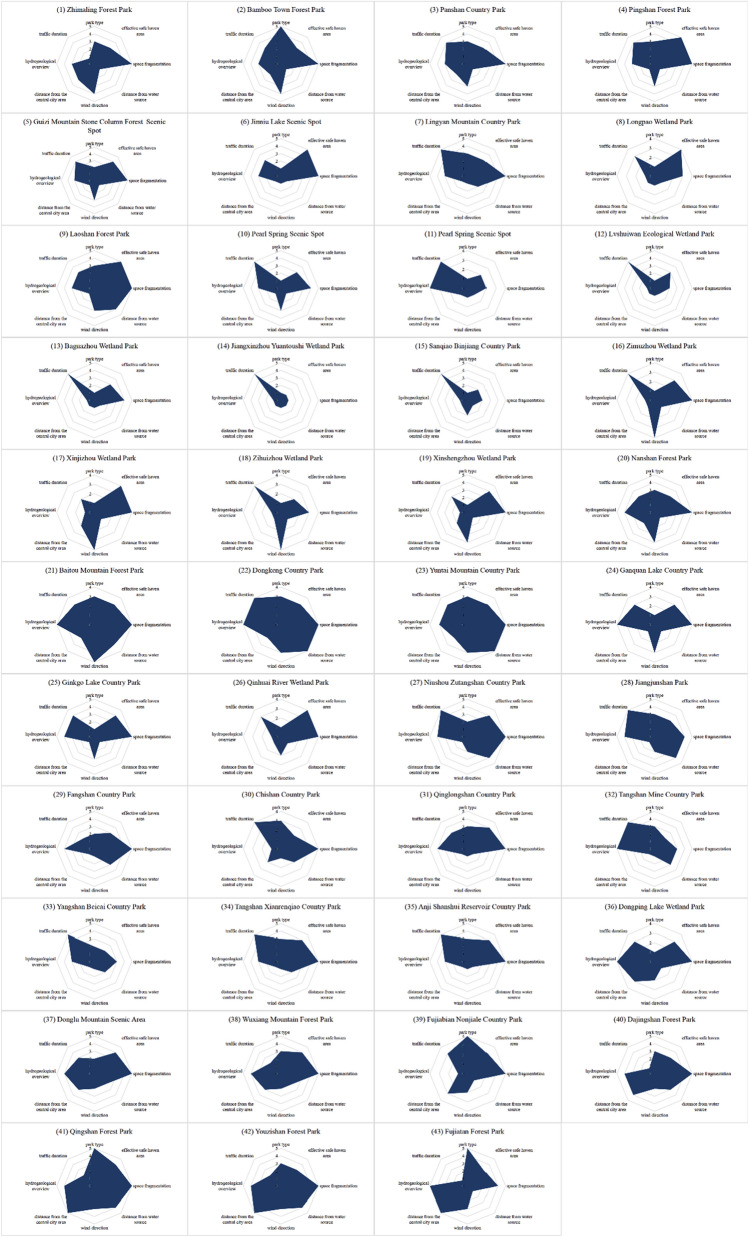
The score distribution of impact factors of country parks.

According to the above impact factor assignment method, each impact factor was assigned to each of the 43 country parks. The results of the assignment show that (Fig. 3): the three impact factors, such as effective avoidance area, spatial fragmentation and traffic length, are superior overall and can provide good prerequisites for the construction of emergency medical facilities; the hydrogeology of the parks differs greatly and the degree of superiority and inferiority is seriously polarized. In addition, the four influencing factors, such as park type, distance from water sources, wind direction where they are located, and distance from the central city, the overall conditions are at a disadvantage, among which the disadvantage of distance from water sources is especially prominent, with 26 country parks straddling water sources or within 200 m from water sources, accounting for 60.5% of the overall number, with a high number and poor distance conditions from water sources. In summary, the 43 country parks in Nanjing have a large overall park scale, high integration, sufficient effective refuge area, and convenient transportation; the parks are located in poor hydrogeological polarization; the parks are of poor type, close to water sources, located in poor wind direction, and close to the central city.

### Park type evaluation

The statistics of country park types (Table 5) show that among the 43 country parks, 16 are Class I, 5 are Class II, 18 are Class III, 1 is Class IV, and 3 are Class V. Nanjing belongs to the Ningzhen-Yang hilly area, with low hills and gentle hills mainly, and low hills, hills and hills together account for 60.8%. In the Implementation Opinions on Strengthening the Construction of Country Parks, it is pointed out that country parks should be set up by natural hills, rivers and lakes, and wetlands, of which the number of country parks built by hills accounts for 50%, and the number of country parks built by water accounts for 41.3%. Therefore, the number of Class III and Class I types of country parks is high. In addition, Nanjing is rich in above- and below-ground heritage resources, so some country park types overlap, for example, 10 country parks such as Tuankou and Dongqiao have both humanistic and mountainous types. Based on the principle of low and strict classification, the country parks with overlapping types were classified as low level for value calculation. Overall, the park types are disadvantageous factors in the site selection process.

**Table 5:** The results of park type assignment of country parks.

Park No	1	2	3	4	5	6	7	8	9	10	11	12	13	14	15
Park type assignment	III	V	III	III	II	I	III	I	III	I	I	I	I	I	I

Park No	16	17	18	19	20	21	22	23	24	25	26	27	28	29	30
Park type assignment	I	I	I	I	III	III	III	III	I	I	I	II	III	II	III

Park No	31	32	33	34	35	36	37	38	39	40	41	42	43		
Park type assignment	III	III	II	III	III	I	II	III	IV	III	V	III	V		

### Evaluation of the effective hedge area of the park

The effective shelter area of the park was counted (Table 6), of which one belonged to Class I, six to Class II, 20 to Class III, 13 to Class IV, and three to Class V. It is worth noting that even the smallest wetland park, Jiangxinzhou Yuantoustone Wetland Park, has an effective refuge area of 17.62 hm^2^, which is close to four times the lower limit of land for emergency medical facilities, so it is clear that the effective refuge area of country parks in Nanjing as a whole is a dominant element.

**Table 6:** Results of effective hedging area assignment of country parks.

Park No	1	2	3	4	5	6	7	8	9	10	11	12	13	14	15
Effective hedge area assignment value	III	III	III	V	IV	V	III	IV	V	III	II	III	III	I	II

Park No	16	17	18	19	20	21	22	23	24	25	26	27	28	29	30
Effective hedge area assignment value	III	IV	II	IV	III	III	III	III	III	IV	IV	IV	III	III	II

Park No	31	32	33	34	35	36	37	38	39	40	41	42	43		
Effective hedge area assignment value	IV	II	II	IV	IV	III	IV	IV	III	III	IV	III	III		

### Evaluation of the Spatial Fragmentation Degree

**Figure 4:**
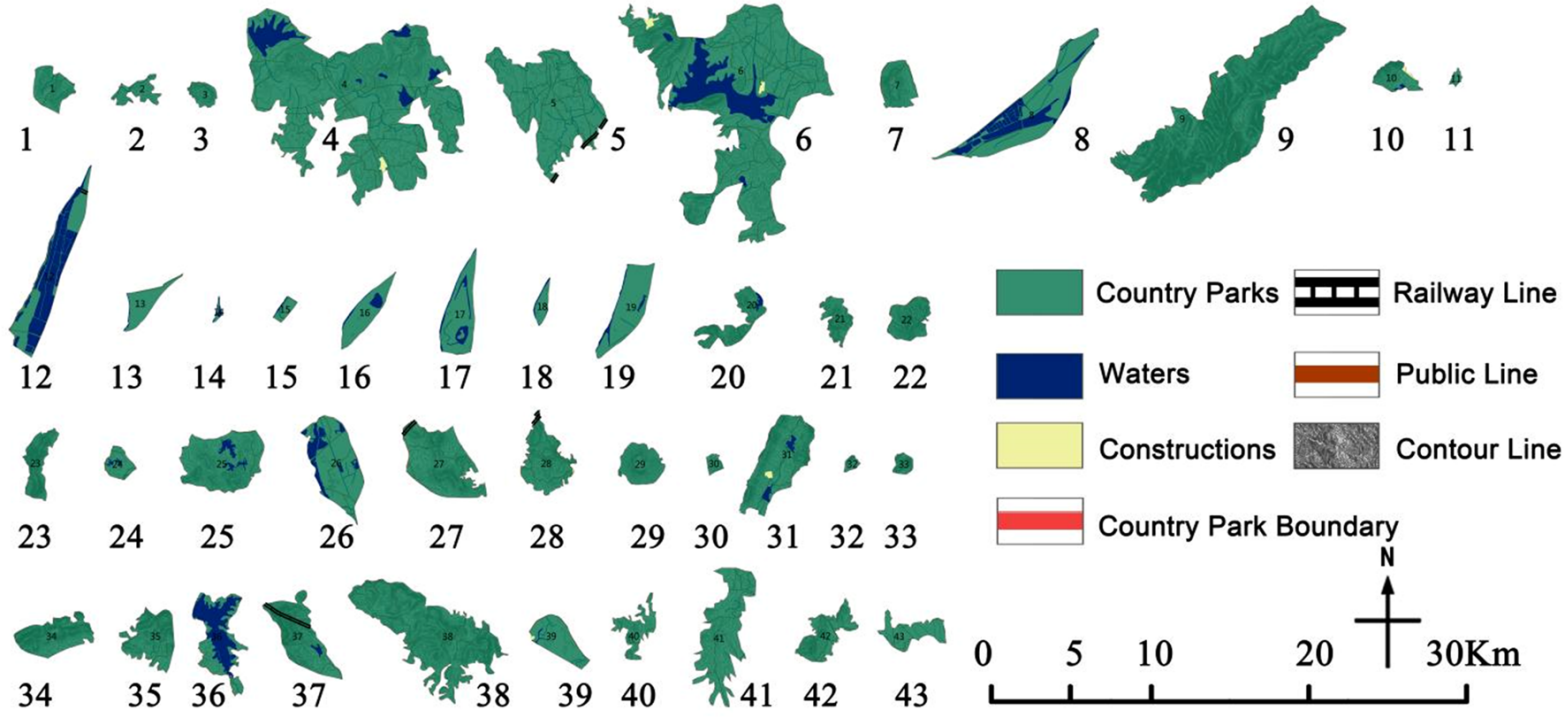
Spatial fragmentation of country parks.

The spatial fragmentation of country parks was counted (Table 7, Fig. 4), of which 1 belonged to Class I, 3 to Class II, 4 to Class III, 14 to Class IV, and 21 to Class V. Overall, the park integration is high and spatial fragmentation is a dominant factor. The statistical results show that the spatial fragmentation and the construction scale as a whole show an inverse trend, that is, the larger the scale the lower the fragmentation, so it is especially harsh for small-scale parks. For example, the Jiangxinzhou Yuantoustone Wetland Park in Jiangxinzhou, the smallest park scale, and contains a large area of wetlands, significantly reducing the effective avoidance area, the fragmentation degree is as high as 0.1703.

**Table 7:** Results of Spatial Fragmentation Degree of country parks.

Park No	1	2	3	4	5	6	7	8	9	10	11	12	13	14	15
Spatial fragmentation assignment	V	V	V	V	V	V	V	III	V	IV	II	II	IV	I	II

Park No	16	17	18	19	20	21	22	23	24	25	26	27	28	29	30
Spatial fragmentation assignment	IV	IV	III	V	V	IV	IV	IV	IV	V	IV	V	IV	V	IV

Park No	31	32	33	34	35	36	37	38	39	40	41	42	43		
Spatial fragmentation assignment	V	III	III	V	V	IV	V	V	IV	V	V	V	IV

### Evaluation of the park distance from the water source area

**Figure 5:**
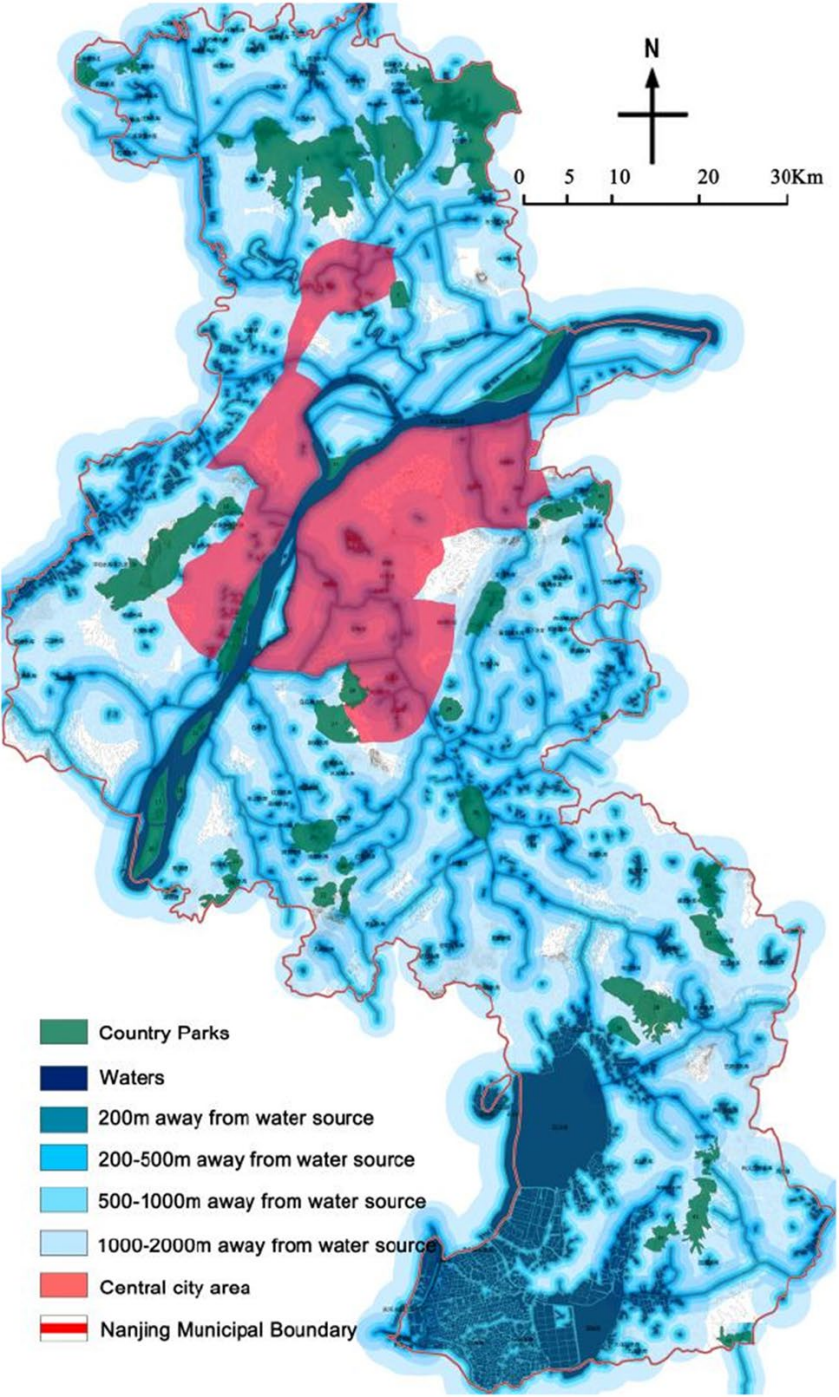
Distances of country parks from water sources (The map was generated using ArcGIS 10.8 (https://www.esri.com/en-us/home) based on China Standard Map (http://bzdt.ch.mnr.gov.cn/)).

Statistics on the distance distribution of parks from water sources (Table 8, Fig. 5) show that 26 parks are located in Class I areas, 6 in Class II areas, 4 in Class III areas, 7 in Class IV areas, and 0 in Class V areas. It is worth drawing attention to the fact that parks located within 200 m of water sources (Class I area) occupy 60.47% of the total, among which 16 country parks, such as Buddha's Hand Lake Country Park, Green Water Bay Wetland Park, and Jinniu Lake Scenic Area, were built directly by rivers, lakes, wetlands, and reservoirs, which is directly related to the setting of country parks in Nanjing relying on rivers, lakes, and wetlands. On the whole, the parks are close to water sources, which is an unfavorable element in site selection.

**Table 8:** Results of Spatial Fragmentation Degree of country parks.

Park No	1	2	3	4	5	6	7	8	9	10	11	12	13	14	15
Distance from the water source area and Value assignment	I	I	I	I	I	I	II	I	IV	I	I	I	I	I	I

Park No	16	17	18	19	20	21	22	23	24	25	26	27	28	29	30
Distance from the water source area and Value assignment	I	I	I	I	I	III	IV	IV	I	I	I	IV	IV	III	II

Park No	31	32	33	34	35	36	37	38	39	40	41	42	43		
Distance from the water source area and Value assignment	I	III	II	II	I	I	II	II	I	III	IV	IV	I

### Wind direction evaluation in the park

**Figure 6:**
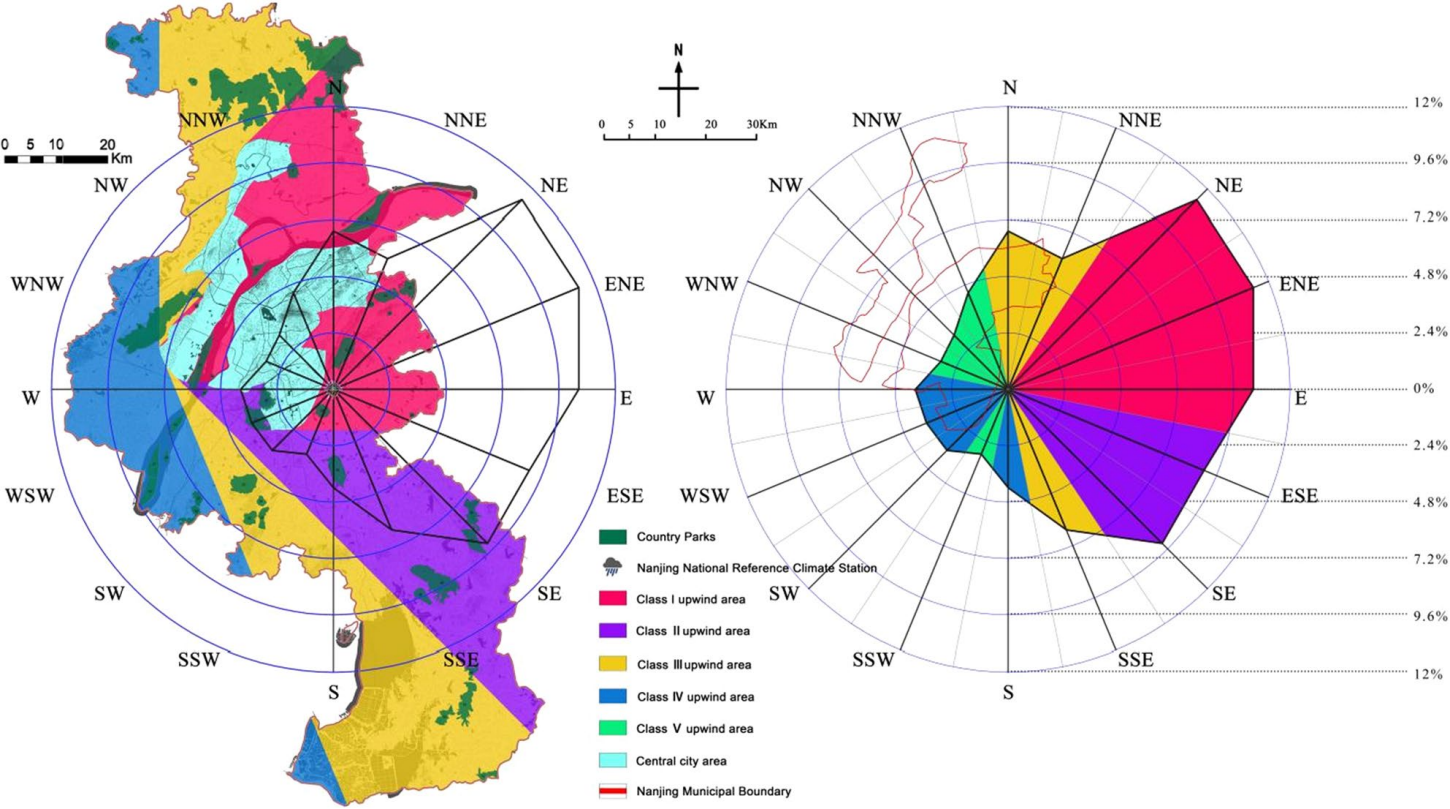
Wind direction distribution in 30country parks (The map was generated using ArcGIS 10.8 (https://www.esri.com/en-us/home) and Adobe Photoshop 2022 (https://www.adobe.com) based on China Standard Map (http://bzdt.ch.mnr.gov.cn/)).

The parks located in the wind direction upwind area were counted (Table 9, Fig. 6), including 14 parks located in Class I area, 9 parks in Class II area, 12 parks in Class III area, 8 parks in Class IV area, and 0 parks in Class V area. The dominant wind direction in Nanjing is more prominent, with northeasterly winds (NE, ENE) prevailing in winter and southeasterly winds (SE, ESE) in summer, and this pattern of concentrated distribution of wind frequency can highlight the degree of superiority and inferiority of the site selection target sites. It is worth noting that when drawing the wind direction of the city, the upwind area is a wind belt with a certain width, and its width is the same as the width of the corresponding angle in the central city. Therefore, the wind bands of sixteen directions centered on the central city have a certain range of overlap, and the principle of low ranking is applied to the overlapping zones. For example, in the southwest-southwest (SSW) wind belt of Nanjing, three wind frequencies of Class IV, Class III and Class II overlap here in turn, then the overlapping part of the Class IV and Class III area is covered by the Class II wind belt. In addition, there are 23 country parks located in Class I and Class II areas, accounting for 53.49% of the total, which is an unfavorable factor in site selection. On the whole, the wind direction located is a disadvantageous factor in the site selection process.

**Table 9:** Results of Spatial Fragmentation Degree of country parks.

Park No	1	2	3	4	5	6	7	8	9	10	11	12	13	14	15
Wind direction Value in the park	IV	IV	III	III	III	I	I	I	III	III	I	I	I	I	II

Park No	16	17	18	19	20	21	22	23	24	25	26	27	28	29	30
Wind direction Value in the park	IV	IV	IV	IV	IV	IV	III	III	III	III	II	II	II	I	I

Park No	31	32	33	34	35	36	37	38	39	40	41	42	43		
Wind direction Value in the park	I	I	I	I	I	II	II	II	II	II	III	III	III

### Park distance from the central city area evaluation

**Figure 7:**
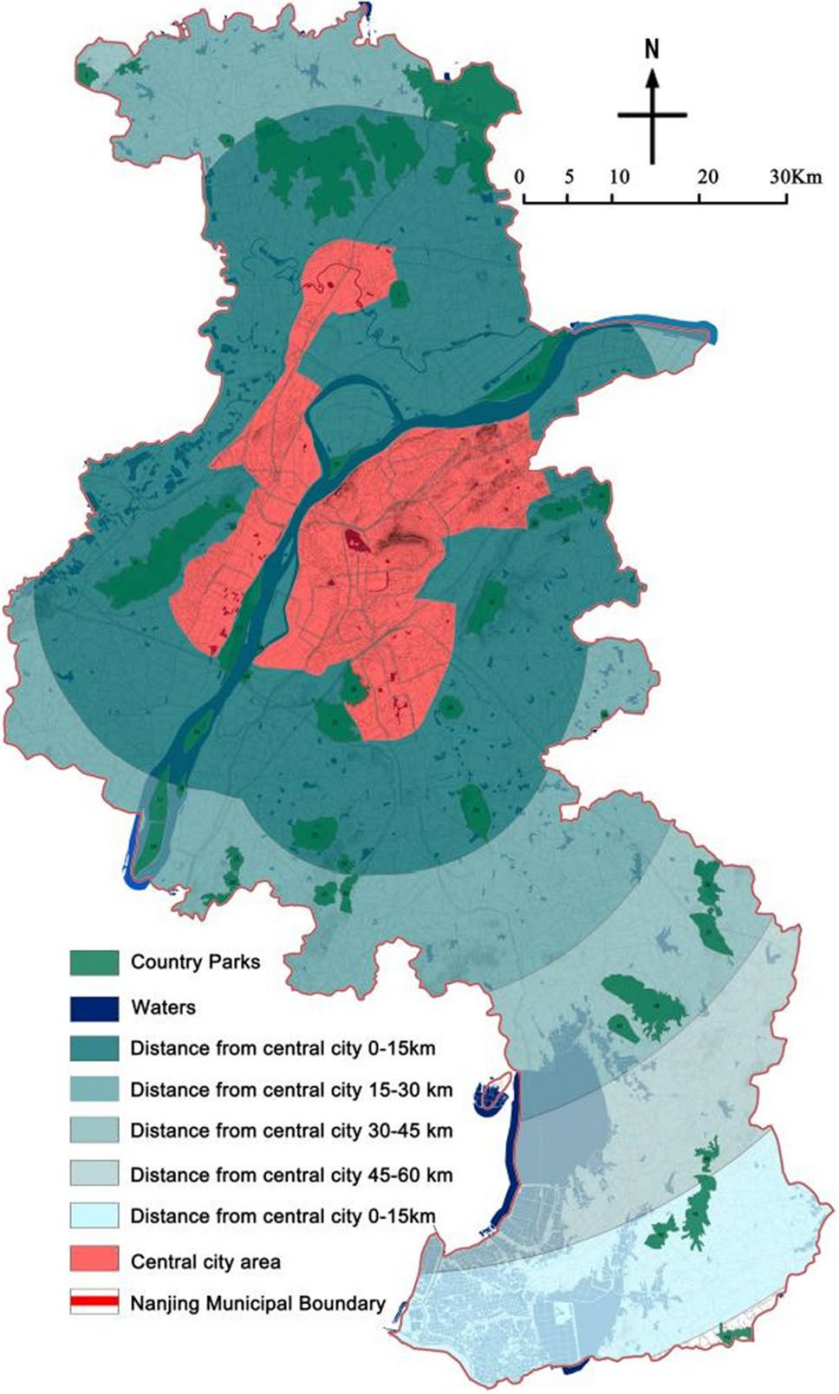
Distance distribution of country parks from the city (The map was generated using ArcGIS 10.8 (https://www.esri.com/en-us/home) and Adobe Photoshop 2022 (https://www.adobe.com) based on China Standard Map (http://bzdt.ch.mnr.gov.cn/)).

The distance of country parks from the central city was counted (Table 10, Fig. 7), of which 25 were located in Class I areas, 9 in Class II areas, 5 in Class III areas, 1 in Class IV areas, and 3 in Class V areas. Among them, 58.14% of the overall number of parks in Class I areas are located closer to the city, which is in line with the principle of country parks serving the life of urban residents and being located and built nearby, but they are not ideal places for siting emergency medical facilities and should be made in advance with corresponding greening isolation measures.

**Table 10:** The results of distance assignment of country parks from the city.

Park No	1	2	3	4	5	6	7	8	9	10	11	12	13	14	15
Assignment of distance from central urban area	III	II	II	I	I	I	I	I	I	I	I	I	I	I	I

Park No	16	17	18	19	20	21	22	23	24	25	26	27	28	29	30
Assignment of distance from central urban area	I	II	I	II	II	II	II	II	I	I	I	I	I	I	II

Park No	31	32	33	34	35	36	37	38	39	40	41	42	43		
Assignment of distance from central urban area	I	I	I	I	I	III	III	III	III	IV	V	V	V

### Hydrogeological general situation evaluation of the park site

**Figure 8:**
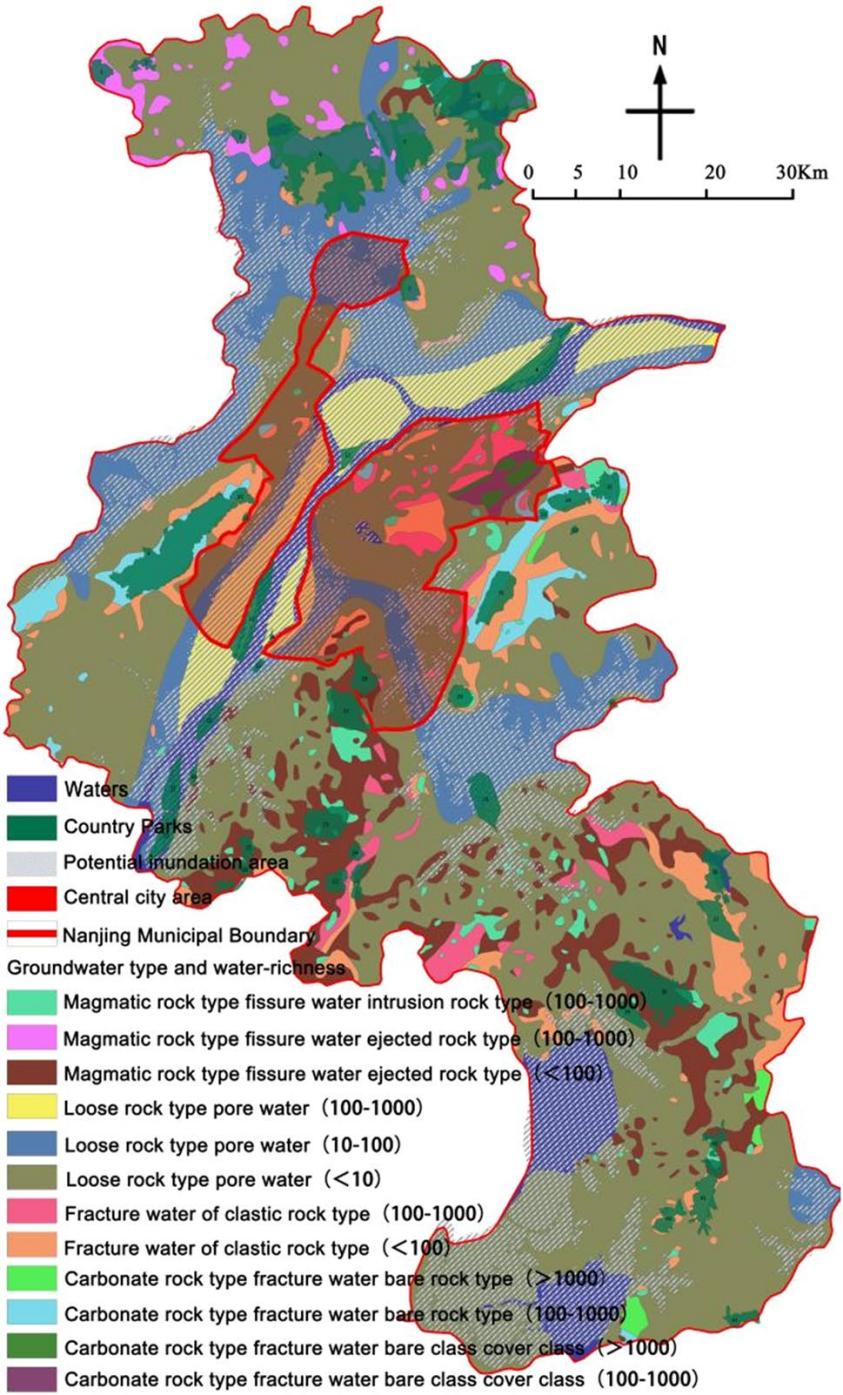
Land type distribution map of country parks (The map was generated using ArcGIS 10.8 (https://www.esri.com/en-us/home) and Adobe Photoshop 2022 (https://www.adobe.com) based on China Standard Map (http://bzdt.ch.mnr.gov.cn/)).

The hydrogeological profiles of the country parks located in the country parks were counted (Table 11, Fig. 8), of which 12 country parks were located in Class I areas, 0 in Class II areas, 13 in Class III areas, 16 in Class IV areas, and 2 in Class V areas. Overall, given that the emergency medical facilities are laid with impermeable layers at the ground level^37^, the depth of groundwater burial, the degree of water enrichment and its permeability have relatively little overall impact on the site selection. However, it is worth drawing attention to the fact that parks in Class I areas have the risk of potential inundation, which is not conducive to epidemic prevention and control and should be carefully selected.

**Table 11:** The results of groundwater depth of 30 country parks.

Park No	1	2	3	4	5	6	7	8	9	10	11	12	13	14	15
Hydrogeological profile assignment	III	III	III	III	III	III	III	I	III	III	IV	I	I	I	I

Park No	16	17	18	19	20	21	22	23	24	25	26	27	28	29	30
Hydrogeological profile assignment	I	I	I	I	IV	IV	IV	III	IV	IV	I	IV	IV	IV	I

Park No	31	32	33	34	35	36	37	38	39	40	41	42	43		
Hydrogeological profile assignment	IV	V	III	III	III	IV	IV	IV	I	IV	IV	IV	V

### Park traffic duration evaluation

**Figure 9:**
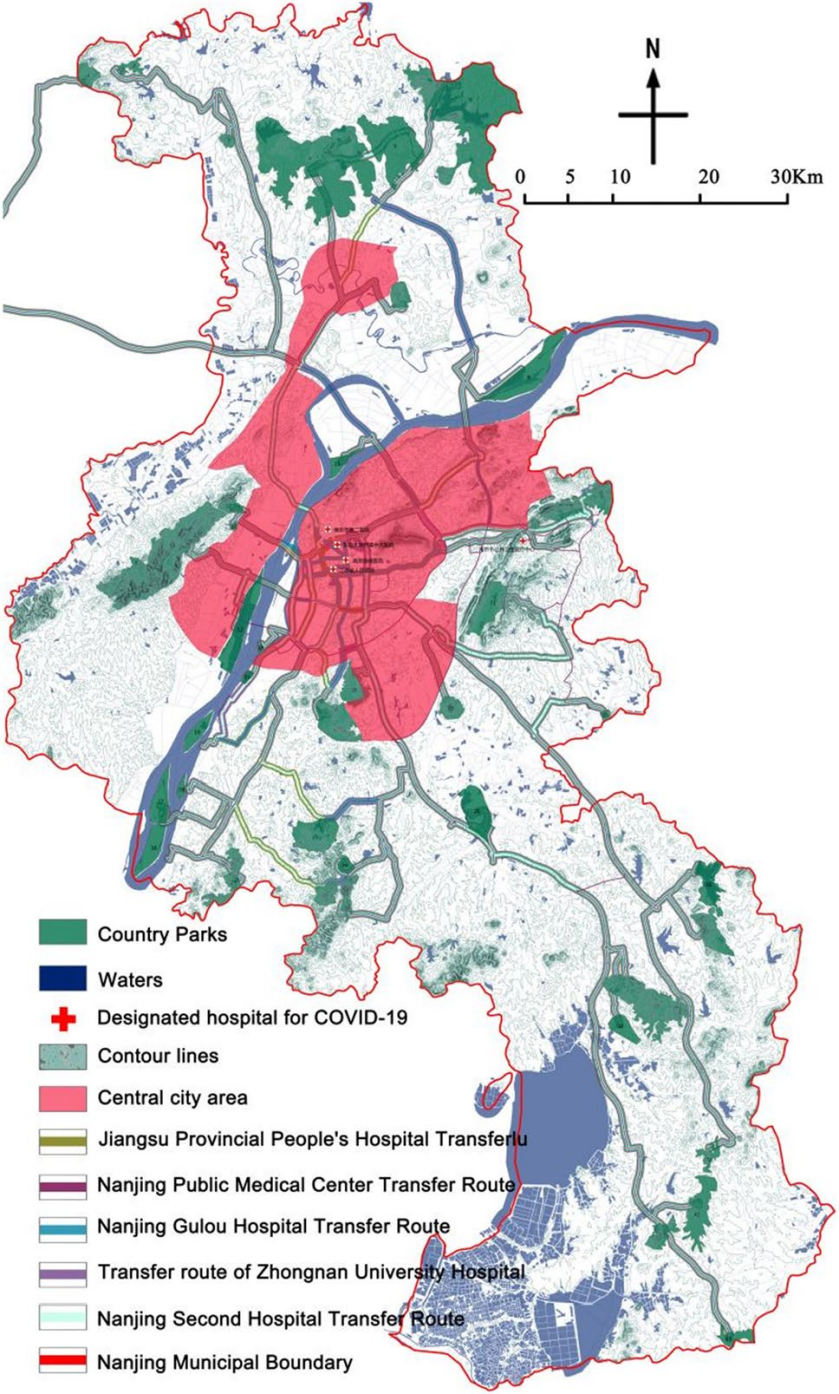
Traffic map of country parks (The map was generated using ArcGIS 10.8 (https://www.esri.com/en-us/home) and Adobe Photoshop 2022 (https://www.adobe.com) based on China Standard Map (http://bzdt.ch.mnr.gov.cn/)).

The transit traffic time was counted (Table 12, Fig. 9), including 3 parks of class I, 5 of class II, 14 of class III, 9 of class IV, and 12 of class V. The results showed that 31 parks were more than 60 min away from the designated infectious disease treatment hospitals, and the longest drive even exceeded 120 min, so the overall traffic length was an unfavorable factor for site selection. It was found that the traffic time and distance were not exactly proportional to each other, and the country parks located in the suburbs of the central city had more traffic nodes and higher traffic flow, so the advantage of traffic time was not particularly prominent.

**Table 12:** The results of traffic length assignment of 30country parks.

Park No	1	2	3	4	5	6	7	8	9	10	11	12	13	14	15
Assignment of traffic duration	I	III	IV	IV	IV	III	V	III	III	V	IV	V	V	V	V

Park No	16	17	18	19	20	21	22	23	24	25	26	27	28	29	30
Assignment of traffic duration	IV	II	IV	III	III	III	IV	III	III	IV	III	V	V	II	IV

Park No	31	32	33	34	35	36	37	38	39	40	41	42	43		
Assignment of traffic duration	III	V	V	V	V	III	III	II	III	I	II	II	I

## Results

**Figure 10:**
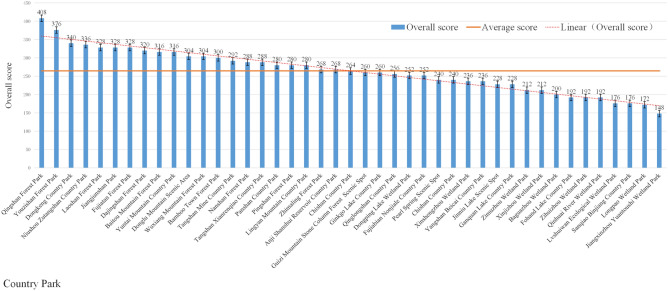
Comprehensive score of country parks.

According to the evaluation results of the above impact factors, the comprehensive scores of 43 country parks are calculated by Formula (4), as shown in Fig. 10:

Among the 43 country parks, Qingshan Forest Park has the highest overall score. This country park is located in Gaochun District is converted from Qingshan Forest Farm, with 92.2% forest coverage, which is a forest type country park with high green coverage, good ecological environment and good forest enclosure, providing a better recreational environment for patients. The effective shelter area of the park is 1602.98 hm^2^, with unified internal land type and high integration, which can provide sufficient expansion space for the construction of emergency medical facilities; in addition, the park is far from the water source, far from the central city, and located downwind all year round, which effectively blocks the spread of virus. In addition, the comprehensive conditions of Youzi Mountain Forest Park and Dongkeng Country Park are also superior, which can be included in the plan together with the construction of emergency medical facilities as alternative sites.

## Discussion

**Figure 11:**
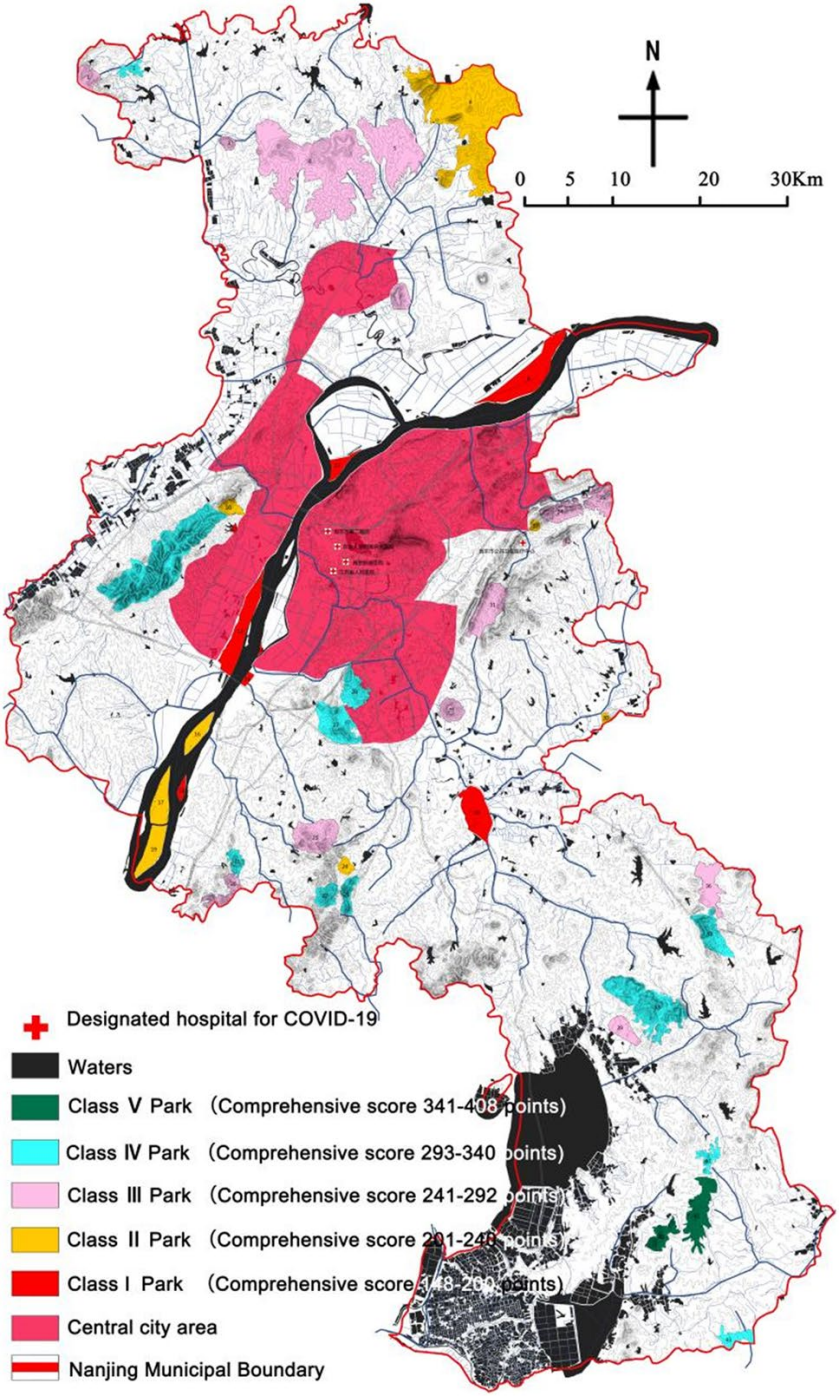
Visualization of the ranking and classification of country parks (The map was generated using ArcGIS 10.8 (https://www.esri.com/en us/home) and Adobe Photoshop 2022 (https://www.adobe.com) based on China Standard Map (http://bzdt.ch.mnr.gov.cn)).

The results of the comprehensive score ranking show that:

1. With a full score of 500, the average score is 264.47, which is at a moderate to low level overall. The highest score is 408 and the lowest score is 148, and the former is 2.76 times higher than the latter, indicating that the assignment method can significantly differentiate the superiority and inferiority of country parks.
2. The parks with good overall ratings are concentrated in Gaochun district in the southeast of the city, and there is an indirect correspondence between the superiority and inferiority of parks and their spatial locations. Among the eight influencing factors, the distance from the central city and traffic time are directly related to the spatial location, while the remaining six influencing factors are very slightly influenced by the spatial location, therefore, the spatial location of the park does not play a decisive role in its overall score (Fig. 11).
3. Among the category V parks with high overall scores, the advantages of the six influencing factors, such as park type, effective risk-avoidance area, spatial fragmentation, distance from water sources, distance from the central city, and hydrogeology, are particularly outstanding. The type of country parks is forest type or forest type with mountainous features; the effective avoidance area of category V parks is 655.46 hm^2^ and 1602.98 hm^2^ respectively, with sufficient land for expansion and high integration; the distance from water sources is far, with the distance greater than 1 km; the distance from the central city is greater than 60 km.
4. The validity advantage of the traffic length influence factor is not obvious, and the time spent is about 1.6 h. The two influence factors of wind direction and traffic duration have no obvious advantages in terms of validity. The above factors are largely restricted by the established conditions, but they can be solved or mitigated by means of greening enclosure and traffic control, and their influence on the site selection is not significant.
5. In view of the fact that different regions are different, the weighting of the influence factors can be adjusted according to local conditions in order to effectively utilize the existing conditions and avoid the unfavorable factors.

In response to the suddenness and strong contagiousness of public health emergencies, we have chosen to use AHP and Delphi methods to construct an evaluation system for emergency medical facility location selection, and to use country parks as backup locations for emergency medical facility construction. The analysis results also strongly support our idea. However, it should be noted that the country parks studied in this article are all built for the purpose of urban residents' leisure and recreation, and their role in combating sudden public health events is rarely considered in planning and design. Therefore, their site selection may not necessarily be the optimal location for constructing emergency medical facilities in the entire urban fringe. Therefore, should we re-examine the types and functions of Urban green space? For example, in the suburbs of cities, we can select suitable areas for construction based on the requirements of emergency medical facility site selection, and then carry out the planning, design, and construction of park green spaces for combating sudden health emergencies in this area. In "normal times", it can provide urban residents with the function of relaxing their mood and releasing stress. During wartime, emergency medical facilities can be quickly established using the park's infrastructure. It can not only alleviate the pressure on the medical system during the outbreak of the epidemic, but also solve the problem of tight urban land use. In this new planning and design, we can fully consider the infrastructure required for the park, such as electricity, water supply, communication, etc., as well as special facilities required for emergency medical facilities, such as isolation areas, temporary wards, etc. In this way, in the event of a public health emergency, the park can quickly transform into an emergency medical facility to provide emergency treatment for infected patients. In addition, for different emergency medical facility needs, we can also classify country parks based on their scale and function. For example, some larger country parks can accommodate more facilities and personnel to cope with major outbreaks of the epidemic. Some smaller country parks can serve as landing or dispersion points for initial response and patient diversion. On this basis, we further consider the uncertainty of the number of infected patients to maximize the admission level of emergency medical facilities, thereby providing a strong and sustainable public health emergency response mechanism for cities to better respond to similar emergencies.

## Conclusion

Under the background of epidemic outbreak, this paper studies the location of emergency medical facilities during the epidemic situation. The Delphi method and AHP method are used to combine the qualitative and quantitative aspects in the research. On the one hand, the opinions of experts can be fully reflected, and on the other hand, the indicators can be further quantitatively analyzed, providing a scientific and reasonable reference basis for emergency management decision-making under the epidemic situation. However, there are still some limitations, which are mainly reflected in the following three aspects:

1. The location of epidemic emergency rescue equipment is different from that of other disaster emergency facilities. The number and location of epidemic emergency facilities are not only limited by distance and demand, but also may consider qualitative factors such as population density and environmental pollution.
2. This study fails to consider the construction cost of emergency medical facilities, procurement cost of emergency resources and transportation cost. On this basis, the next study will consider the minimization of comprehensive cost, optimize the location of emergency facilities, and reasonably plan the number of allocated medical resources and the route of transporting infected patients.
3. Due to the great differences in conditions in different countries and regions, various influencing factors have different degrees of influence on the location of emergency medical facilities. Therefore, researchers should increase or decrease the types of influencing factors according to the situation of the study place, and adjust the weight according to the degree of influence, so as to give play to the favorable conditions of the target area and avoid the unfavorable factors.

## Data Availability

The datasets and analyed during the current study are available from the corresponding author on reasonable request.
